# The full blood count as a tool of differentiation between benign and malignant lymphadenopathy in pediatric and adolescent populations

**DOI:** 10.1016/j.jped.2025.05.001

**Published:** 2025-06-01

**Authors:** Mariana Moreira Magnabosco da Silva, Leniza Costa Lima Lichtvan, Thaís Cugler Meneghetti

**Affiliations:** a Complexo Hospital de Clínicas da Universidade Federal do Paraná, Curitiba, PR, Brazil; b Serviço de Hemato-Oncologia Pediátrica do Complexo Hospital de Clínicas da Universidade Federal do Paraná, Curitiba, PR, Brazil; c Hospital de Clínicas da Universidade Federal do Paraná (UFPR), Curitiba, PR, Brazil

**Keywords:** Lymphadenopathy, Lymphoma, Full blood count, Pediatrics, Diagnostic tests

## Abstract

**Objective:**

Studies have shown the benefit of ratios between cell types in full blood count for the diagnosis and prognosis of various pathologies, but its use in the investigation of lymphadenopathy is still controversial. The aim of this study is to assess whether there is a difference between the full blood count ratios of children and adolescents with lymphadenopathy who had benign or malignant causes identified in excisional biopsies during the investigation of lymphadenopathy.

**Method:**

This is a cross-sectional observational study. A total of 72 participants between 0 and 17 years old who underwent an excisional lymph node biopsy and had their full blood count collected up to 15 days before the procedure were included. The participants were divided into two groups, malignant and benign, according to the biopsy result. Neutrophils/lymphocytes (N/L), monocytes/lymphocytes (M/L), and platelets/lymphocytes (P/L) ratios were then calculated and compared between the groups.

**Results:**

The mean full blood count ratios for the benign and malignant groups, respectively, were: N/L 2.36 × 3.28 *(p=0.09)*, M/L 0.30 × 0.32 *(p=* 0.722*)*, P/L 180.53 × 191.30 *(p=0.249)*.

**Conclusion:**

The use of full blood count ratios as predictors of malignant or benign results has no proven statistical significance. Higher ratio values tend towards malignant results but should be interpreted with caution, as this is only one of the factors to be considered during the investigation.

## Introduction

Lymphadenopathy is a common complaint amongst pediatric patients and is defined as an increased lymph node with altered characteristics, such as appearance, consistency on palpation, and relationship with adjacent tissues. This condition can have many etiologies: infectious, immunological, endocrine, lymphoproliferative, medicinal, and neoplastic. In children, most peripheral lymphadenopathies have a benign and self-limiting cause, which can usually be identified through anamnesis and physical examination. The biggest predicament happens when the cause cannot be identified even with complementary tests and the symptom remains for more than four weeks. The main concern is the exclusion of malignant causes, such as lymphoma.^1^

The gold standard test for lymphoma diagnosis is a histopathological analysis of the material obtained through an excisional biopsy, which allows a complete examination of the architecture of the lymph node. However, this is an invasive procedure, subject to inherent risks, as well as to those related to the use of general anesthesia.^2^ Therefore, an excisional biopsy should not be indicated for all cases of peripheral lymphadenopathy without a defined cause. At the same time, failing to recommend a biopsy can delay the diagnosis and treatment of lymphoma, worsening the prognosis.^3^

Fine needle biopsy of the lymph node, despite being a minimally invasive option in adults, ends up having the same morbidity in children, since it is unlikely that a child will cooperate with the puncture, requiring sedation and analgesia in an environment with adequate support. In addition, this technique allows only cytological analysis, being more useful for collecting cultures than for histopathological analysis. A fine needle puncture without alterations does not exclude malignancy, since the puncture may have been performed in a healthy area of ​​the lymph node. In addition, if there is an alteration suggestive of lymphoma, an excisional biopsy is mandatory for the diagnosis and classification of the neoplasia. Thus, fine needle biopsy in children is a procedure with the same operational complexity as an excisional biopsy and contributes little to the etiology of lymph node enlargement.^4^^,^^5^

The full blood count is a rapid, objective, widely accessible, and low-cost diagnostic tool. In addition to the absolute and relative concentrations of cellular types present in the peripheral circulation, relationships between different cell types have been researched, aiding in the diagnosis and prognosis of various diseases.^6^ The ratios most mentioned in the literature are neutrophils/lymphocytes (N/L), monocytes/lymphocytes (M/L), and platelets/lymphocytes (P/L). In the tumor microenvironment, each cell type interferes in oncogenesis in a different way. Lymphocytes induce the apoptosis of tumor cells, therefore lymphopenia enables the escape of these cells, facilitating metastasis and recurrences.^7^ Neutrophils secrete oncogenic interleukins. Monocytes are markers of high tumor loads, stimulating the proliferation and migration of tumor cells, as well as genetic instability. Platelets, when activated, perpetuate the inflammatory cascade.^7^^,^^8^

Recent studies sought to assess the utility of the ratios obtained through the full blood count in differentiating patients who have received benign or malignant diagnoses through excisional lymph node biopsies. Çolak et al.^5^ evaluated 46 participants and concluded that the N/L ratio is higher in patients with Hodgkin's lymphoma. Kamiya et al.^3^ evaluated 256 participants and concluded that the M/L ratio is higher in patients with lymphoma. However, in both studies, only adults were included in the samples. Tezol et al.^1^ observed that, in a sample of 190 children and adolescents, patients diagnosed with malignancy had higher N/L, M/L, and P/L ratios. However, cases of granulomatosis, histiocytosis, and reactive lymphadenopathy that had received antibiotic therapy before the biopsy were excluded from that study, ruling out the applicability of this diagnostic tool in daily practice. Thus, more research is needed to clarify whether full blood count can be a tool for differentiating benign and malignant causes of lymphadenopathy in children, avoiding unnecessary surgical procedures and without delaying the diagnosis of lymphomas.

This study aims to assess whether there is a difference between the ratios of N/L, M/L, and P/L in full blood count of children and adolescents who have had benign or malignant causes identified in excisional biopsies during an investigation of lymphadenopathy.

## Methods

This is an analytical cross-sectional study with retrospective data collection, approved by the institution's Research Ethics Committee.

Initially, the present study evaluated 198 participants under the age of 18 who had undergone excisional lymph node biopsy at a tertiary public university hospital between 2011 and 2023 for a lymphadenopathy investigation. Within this group, the following were excluded: histopathological analysis of lymph nodes for staging solid tumors (*n* = 58); those who underwent surgery that resulted in timely lymph node excisions, such as acute abdomen and elective splenectomy (*n* = 25); histopathological analysis reports that were uncertain as to benignity (*n* = 10); diagnosis of relapsed leukemia (*n* = 1); and participants with no full blood count available up to 15 days before the biopsy (*n* = 32). Accordingly, the final sample comprised 72 participants.

Full blood count collected externally was excluded from the samples to maintain the uniformity of the analysis since all full blood count samples from the department are submitted to a microscopic analysis of the blood smear slide, in order to verify the automated results.

Data such as date of birth, gender, age at the date of the biopsy, date of the biopsy, date of the full blood count, days between the collection of the full blood count and the biopsy, and description of the result of the histopathological analysis were collected from each patient's electronic medical record, along with the following full blood count parameters: erythrocyte count, hemoglobin, mean corpuscular volume (MCV), red cell distribution width (RDW), leukocytes, basophils, eosinophils, band cells, neutrophils, lymphocytes, monocytes, platelets, and mean platelet volume (MPV). White cells were considered as an absolute count. The N/L, M/L, and P/L ratios were calculated using the Microsoft Excel 365 software. According to the histopathological analysis of the biopsy, participants were divided into two groups (benign and malignant) for statistical comparison.

The gender homogeneity in both groups defined by the biopsy result was evaluated by the Chi-Square test. The quantitative variables of participants with benign and malignant results were compared by the Mann-Whitney non-parametric test (or the Student's *t*-test for independent samples). By calculating the area below the curve, the ROC curve was fitted to evaluate the diagnostic power of the N/L, M/L, and P/L variables. Values of *p* *<* *0.05* implied statistical significance. Data were analyzed using the Stata/SE v.14.1 computer program (StataCorpLP, USA).

The reference values presented by Moosmann et al., [6] according to age group and gender, were used to verify the normality of the N/L and P/L ratios. The M/L ratio was not evaluated due to the absence of published reference values. Results under 2.5% were considered below the reference value, those between 2.5% and 97.5% were considered within the reference value, and those over 97.5 % were considered above the reference value.

## Results

Of the 72 participants, 43 received a benign biopsy result and therefore were placed in the benign group, while 29 had a malignant result and were categorized under the malignant group. The prevalence of malignancy in the sample was 40.3%. Among the diagnoses considered benign, the following were reported: granulomatous disease (*n* = 9), nonspecific changes of reactive appearance (*n* = 30), fungal infection (*n* = 2), histiocytosis (*n* = 2), areas of necrosis, and suppuration (*n* = 10). Some reports identified more than one benign finding in the same sample.

Table 1 exhibits the similarity within gender, age, and interval since the full blood count between groups. Regarding absolute full blood count values, no statistically significant difference between the two groups evaluated was found.

**Table 1:** General sample characteristics and hematological parameters.

	Benign group (*n* = 43)	Malignant group (*n* = 29)	*P* value^a^
**General characteristics**			
Gender – Frequency	Female = 34.9%	Female = 41.4%	*p* = 0.577
	Male = 65.1%	Male = 58.6%	
Age (years) – Average	8.26 (0.60–16.30)	8.65 (0.90–17.90)	*p* = 0.721
Time between full blood count and biopsy (days) – Average	3.53 (0–15)	3.14 (0–14)	–
**Full blood count parameters**			
Erythrocytes (x10^6^) – Average	4.27 (±0.70)	4.49 (±0.58)	*p* = 0.198
Hemoglobin (mg/dl) – Average	11.1 (±2.22)	11.3 (±1.83)	*p* = 0.718
MCV (fL) – Average	79.2 (±12.54)	76.1 (±5.99)	*p* = 0.228
RDW ( %) – Average	15.6 (±3.90)	15.2 (±2.66)	*p* = 0.735
Leukocytes – Average	10,125 (±5,863)	10,440 (±7,080)	*p* = 0.787
Basophils – Median	0 (0–146)	0 (0–122)	*p* = 0.261
Eosinophils – Median	165 (0–5124)	140 (0–1197)	*p* = 0.462
Band cells – Median	0 (0–2620)	110 (0–4,732)	*p* = 0.473
Neutrophils – Average	5,270 (± 3527)	6,689 (±5,055)	*p* = 0.200
Lymphocytes – Median	2,615 (115–95,630)	2,227 (500–16,796)	*p* = 0.230
Monocytes – Median	560 (60–5310)	600 (0–2,704)	*p* = 0.770
Platelets – Average	348,206 (±196,675)	331,724 (±118,750)	*p* = 0.877
MPV (fL) – Average	9.4 (±2.11)	9.7 (±1.36)	*p* = 0.514

a Mann-Whitney non-parametric test; *p* < 0.05. MCV, mean corpuscular volume; RDW, red cell distribution width; MPV, mean platelet volume.

**Figure 1:**
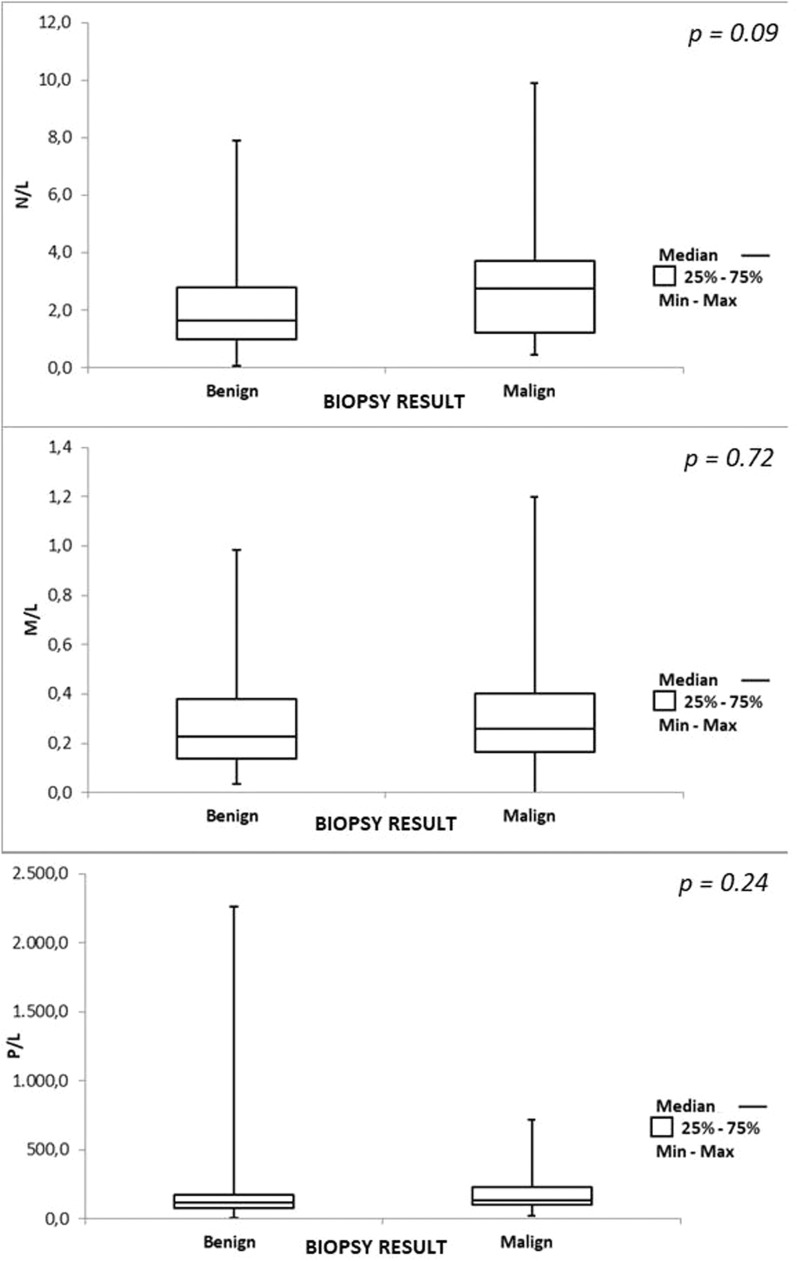
Box plot of full blood count ratios.

Figure 1 illustrates the results of the N/L, M/L, and P/L ratios. There was no difference in the ratios between the groups.

Limits were established to maximize discernment between benign and malignant etiologies. Values above the limit are associated with malignancy, while values below it are related to benignity. The indices that represent performance as diagnostic tests are shown in Table 2.

**Table 2:** Full blood count ratio indicators.

	Sensitivity	Specificity	Accuracy	Limit	PPV	NPV
N/L	58.6%	67.4%	63.9%	2.235	54.8%	70.7%
M/L	55.2%	55.8%	55.6%	0.238	45.7%	64.9%
P/L	48.3%	69.8%	61.1%	155.47	51.9%	66.7%

N/L, neutrophils/lymphocytes; M/L, monocytes/lymphocytes; P/L, platelets/lymphocytes; PPV, positive predictive value; NPV, negative predictive value.

**Figure 2:**
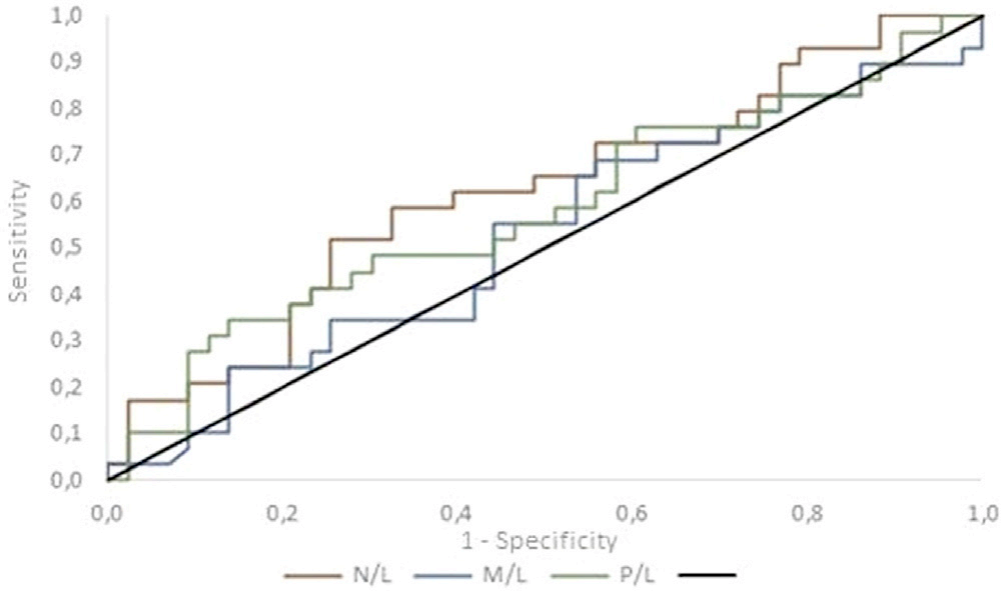
ROC curves of full blood count ratios. N/L, neutrophils/lymphocytes; M/L, monocytes/lymphocytes; P/L, platelets/lymphocytes.

Figure 2 compares the ROC curves for each ratio. The null hypothesis that the ratios have no power to discriminate between benign and malignant cases (area under the curve = 0.5) was tested versus the alternative hypothesis that the ratios have the power to discriminate between benign and malignant cases (area under the curve > 0.5). The area under the ROC curve was estimated as: N/*L* = 0.618 (95 % confidence interval [CI]: 0.485–0.752); M/*L* = 0.525 (95 % CI: 0.387–0.663) and P/*L* = 0.581 (95 % CI: 0.442–0.719). These results do not provide evidence for the rejection of the null hypothesis (*p* = 0.090).

Considering the reference values published by Moosmann et al., [6] no statistically significant difference for the N/L (*p* *=* 0.121) and P/L (*p* = 0.102) ratios were found in those groups. Therefore, values above the reference range are not associated with a malignant result in the biopsy.

## Discussion

In the present study, the N/L, M/L, and P/L ratios did not differ between cases of benign and malignant lymphadenopathy in children and adolescents.

Most cases of lymphadenopathy have their etiology determined by clinical history, physical examination, and ancillary tests, but the most challenging ones may remain uncertain and require the gold standard test, that is, the excisional biopsy of the lymph node, to verify malignancy. The highest prevalence of lymphoma usually occurs from the age of 7,^9^ and corroborating the literature, an average of 8 years of age was found in the sample of patients that required further investigation of lymphadenopathy in the present study.

Although mandatory for diagnosing malignancies, every biopsy resulting in reactive lymphadenopathy is deemed as unnecessary surgical intervention^5^ as surgeries impose a stressful experience for pediatric patients and their parents,^10^ and are also susceptible to complications.

Edomwonyi et al.^11^ evaluated children and adolescents who underwent surgical procedures under general anesthesia. The incidence of intraoperative complications (cardiovascular and respiratory) was 9.3%, and postoperative complications (cardiovascular and pain) was 14.8%. Specifically regarding the excisional lymph node biopsy procedure, Bassiouni et al.^2^ found a complication rate of 2.5% and Campanelli et al.^12^ a rate of 12.7%. Complications included cervical hematomas, seromas, transient paresis of the marginal mandibular nerve, abscesses, and wound dehiscence. It is evident that excisional lymph node biopsy is a safe, low-risk procedure, presenting low mortality and morbidity rates.^13^ However, the procedure must be appropriately indicated, given its potential for complications, family and psychosocial stress, and overload of healthcare systems.

The use of biomarkers for diagnostic and prognostic purposes has been increasing. In the context of investigating lymphadenopathy, a new biomarker could indicate the need for a biopsy more precisely, avoiding unnecessary procedures without delaying the prognosis of malignancy. Studies have been published on serum markers that are highly predictive of lymphoma and refer to biopsies more accurately, such as soluble interleukin 2 receptor and serum thymidine kinase, although they are expensive and not widely available in practice.^5^

The full blood count is a broadly accessible, cost-effective, objective, and rapid tool. However, its full potential in daily practice has yet to be fully explored. In addition to absolute and relative concentrations of cellular types, ratios between different cell types have been the subject of research, aiding in the diagnosis and prognosis of various diseases across diverse medical specialties.^6^

Malignant tumors are surrounded by matrix and stromal cells, as well as the vascular and lymphatic network. These elements constitute the tumor microenvironment, which is closely linked to each stage of tumoral genesis. Hematological neoplasms are characterized by a microenvironment that is slightly different from solid tumors.^7^

Lymphocytes are highly prevalent in the histopathology of lymphoma, and elevated intratumoral levels often predict a better outcome. The interaction between CD4+ and CD8+ is important for inducing tumor cell apoptosis. A low number of infiltrating lymphocytes in the tumor microenvironment may facilitate metastasis and recurrences. The presence of lymphopenia usually reflects severity and favors the escape of tumor cells from lymphocyte action.^7^^,^^14^ Lymphocyte count mirrors the body's stress levels: the higher the stress, the lower the lymphocyte count.^8^

Although neutrophils undeniably provide benefits in the context of infection and trauma, their role in oncogenesis is problematic. Despite exhibiting anti-tumoral activity, neutrophils also suppress cell destruction by immune cells and secrete multiple cytokines, such as interleukin-2, interleukin-10, and TNF-α, which promote tumoral genesis. This could be the reason why more than15% of malignant neoplasms are triggered by infections.^7^ The more severe the inflammatory reaction, the higher the absolute neutrophil count.^8^

Monocytes play an important role in the microenvironment and should be considered as markers of high tumor load. Tumor-associated macrophages stimulate tumor cell proliferation, migration, and genetic instability, and promote the proliferation of blood and lymphatic vessels, which facilitates metastasis. In lymphomas, a higher density of intratumoral macrophages is associated with disease progression and a worse prognosis.^7^

Platelets have a leading role both in the initial stage and in inflammation and immune response regulation, and can be considered perpetuators of the inflammatory cascade.^15^ Once the antigen is detected, platelets are rapidly activated and begin to control the inflammatory response, directly regulating the activities of neutrophils, lymphocytes, and the capacity of endothelial cells, promoting the aggregation of various adhesion molecules and cytokines of tissue damage.^8^^,^^16^

Regarding simple full blood count parameters, the present study found no difference between the groups. Kamiya et al.,^3^ Tezol et al.,^1^ and Çolak et al.^5^ found lower hemoglobin levels in the group of patients with malignancy. Çolak et al.^5^ also found a higher absolute number of neutrophils. Tezol et al.^1^ identified higher RDW values in the group with malignancy, although the difference was not maintained after multivariate analysis.

The N/L ratio has been studied as a prognostic marker in infectious diseases, postoperative complications, and oncological diseases, and is a predictor of mortality in cardiovascular diseases,^6^ as well as a marker of generalized inflammation.^1^^,^^17^^,^^18^^,^^19^ A high N/L ratio suggests a poor prognosis in hematological neoplasms, since it increases at the expense of an increase in neutrophils or a reduction in lymphocytes, shifting the balance towards the pro-tumor inflammatory state. Thus, an increase in N/L indicates a worse prognosis.^5^^,^^20^^,^^21^ Patients diagnosed with lymphoma presented a tendency to have higher N/L values than patients with benign biopsy results. Tezol et al.^1^ found similar data, but the significance was not maintained after multivariate analysis. Çolak et al.^5^ demonstrated this relationship with higher values in adult patients with Hodgkin's lymphoma compared to adult patients with benign biopsy results. In the present study, regarding the reference values, 30.2% of the benign group had N/L values above the reference range, compared to 48.3% of the malignant group, although this sample had no statistical significance.

The M/L and P/L ratios had no statistical relevance in this study as a diagnostic test. Kamiya et al.^3^ found higher M/L ratio values in adults with a diagnosis of lymphoma when compared to adults with a benign diagnosis, with a 0.42 limit, while in the present study, the most effective limit would be 0.28. Considering histopathology, this is a plausible result, since a higher number of monocytes and a lower number of lymphocytes result in a higher numerical value of the ratio and are pro-tumor cell alterations. In their study, Tezol et al.^1^ found statistically significant relationships both in children with lymphoma and in those with benign outcomes, but they lost significance after the multivariate analysis. In this study, 23.3% of the benign group had P/L values above the reference values, compared to 41.4% of the malignant group, resulting in no significant difference.

Significant heterogeneity between the sample in this study and those of other authors was observed. The first difference found in the samples was the prevalence of malignancy. The present study found a prevalence of malignancy of 40.3%, a higher rate than that reported in the literature, between 13% and 27%.^1^ This difference may be explained by selection bias when obtaining the patient sample from a tertiary service, to which patients are referred for further investigation since the history, physical examination, and complementary tests available were not sufficient to rule out malignancy. Several patients already have a benign etiology defined in primary care and do not even reach the tertiary service. Secondly, the inclusion and exclusion criteria are quite varied, as well as differences in the age range, period of full blood count collection, antibiotic use, and ethnicity of the participants.

Kamiya et al.^3^ and Çolak et al.^5^ only analyzed adults and did not disclose the interval between the full blood count and biopsy dates. The pathologies included in the benign and malignant groups of Kamiya et al.^3^ are very similar to those in the present study. Çolak et al.^5^ excluded granulomatosis from the benign group and included only Hodgkin's lymphoma in the malignant group. Tezol et al.^1^ analyzed only children and adolescents, as did this study. All the participants had their full blood count taken two days before the biopsy, while this interval ranged from zero to fifteen days in the present research. The benign group differs greatly from ours in that it excludes cases of granulomatosis, histiocytosis, and any reactive lymphadenopathy that had received antibiotic therapy up to 15 days before the biopsy.

The main limiting factors of the present study were: sample size and selection bias, lack of knowledge about the comorbidities of the participants; and the liability of the full blood count, which could be influenced by other stressors up to 15 days before the biopsy. An expansion of the study sample and a meta-analysis for a systemic analysis of published studies on the subject are recommended for future research.

In conclusion, there was no difference between the full blood count ratios of children and adolescents who had benign or malignant causes identified by excisional biopsy during the investigation of lymphadenopathy. Therefore, the use of full blood count ratios as a predictor of malignant or benign outcomes does not have enough proven statistical significance to be incorporated into daily practice. Given the literature presented, higher ratio values tend towards malignant results, but they should be interpreted with caution, emerging as only one of the factors to be considered during the investigation.

## Funding

The authors themselves.

## Conflicts of interest

The authors declare no conflicts of interest.
